# Localization of Mixed Completely and Partially Polarized Signals with Crossed-Dipole Sensor Arrays

**DOI:** 10.3390/s151229894

**Published:** 2015-12-17

**Authors:** Kun Wang, Jin He, Ting Shu, Zhong Liu

**Affiliations:** 1 Department of Electronic Engineering, Nanjing University of Science and Technology, Nanjing 210094, China; wk_1024@163.com (K.W.); eezliu@mail.njust.edu.cn (Z.L.); 2 Shanghai Key Laboratory of Intelligent Sensing and Recognition, Department of Electronic Engineering, Shanghai Jiaotong University, Shanghai 200240, China; tingshu@sjtu.edu.cn

**Keywords:** electromagnetic wave, dipole sensor, partially polarized signal, complete polarized signal, polarization classification

## Abstract

In this paper, we investigate the problem of source localization and classification under the coexistence of both completely polarized (CP) and partially polarized (PP) electromagnetic (EM) signals, using a crossed-dipole sensor array. We propose a MUltiple SIgnal Classification (MUSIC)-based solution, which does not require multidimensional searches. Moreover, the proposed method need no estimation of the degree of polarization of signals. The efficacy of the proposed method is examined by comparing with existing methods.

## 1. Introduction

Estimation of arrival angles of multiple narrowband electromagnetic (EM) planewave signals is a key problem in many engineering applications including radar, wireless communications and seismic exploration. During the past decades, many effective methods have been proposed. These methods include MUltiple SIgnal Classification (MUSIC)-based algorithms [1,2,3], Estimation of Signal Parameter via Rotational Invariance Technique (ESPRIT)-based algorithms [4,5,6,7], subspace fitting based method [8], propagator-based method [9], and vector cross product based algorithms [10,11,12]. In addition, [13,14,15] proposed methods for EM source localization with various triad compositions, [16,17] studied the electromagnetic vector sensor direction finding in the presence of coherent signals, and [18] considered the EM source localization for Multiple-Input Multiple-Output (MIMO) array applications.

All the above mentioned algorithms are based on the assumption that the impinging signals are complete polarized (CP). However, in applications such as ionospheric and radar, signals are often partially polarized [19,20]. For example, even though the original radar transmitted signal is completely polarized, the polarization states of the reflected signals may vary with time due to the nonstationary behavior of targets, clutter, and interferences [19]. Unlike the CP signals, where each has invariant polarization, the states of polarization of the PP signals vary with time, and hence, the algorithms designed for CP signals are not applicable to the PP signals.

Investigation on partially polarized scenario can be found in [21,22,23]. [21] derived a maximum likelihood (ML)-based algorithm with a cross-dipole array. [22] developed an ESPRIT-based algorithm with a six-component EM vector sensor array. [23] considered the partially polarized signals be located in near-field, and presented the ML- and subpace-based algorithms. These algorithms are not suitable for CP signals without the prior information on the degree of polarization (DOP) of the signals. In fact, in practical applications, where the prior knowledge on the signals’ degree of polarization are always unavailable, it is more realistic to assume that some signals are completely polarized while the others are partially polarized. Although algorithms for angle estimation of CP signals or PP signals have been investigated intensively, little work [24] has been done to deal with the problem of direction finding under the coexistence of both CP and PP signals.

In this paper, we propose a conventional MUSIC-based solution to the angle estimation and signal classification problem under the coexistence of both CP and PP signals. We define two estimators, which are termed as CP and PP estimators, to obtain the angle estimates of the CP and PP signals separately. We also introduce a simple but efficient method for discriminating the CP and PP signals. Finally, we present the simulation results to examine the efficacy of the proposed method by comparing with two existing methods in [21,24] and with the CRB.

Throughout the paper, transpose, conjugate transpose, complex conjugate and pseudo inverse are denoted by superscripts *T*, *H*, *, and †, respectively. ⊗ represents the Kronecker-product operator, $I_{m}$ stands for a m×m identity matrix, and $0_{m,n}$ is a m×n zero matrix.

## 2. Signal Model

**Figure 1:**
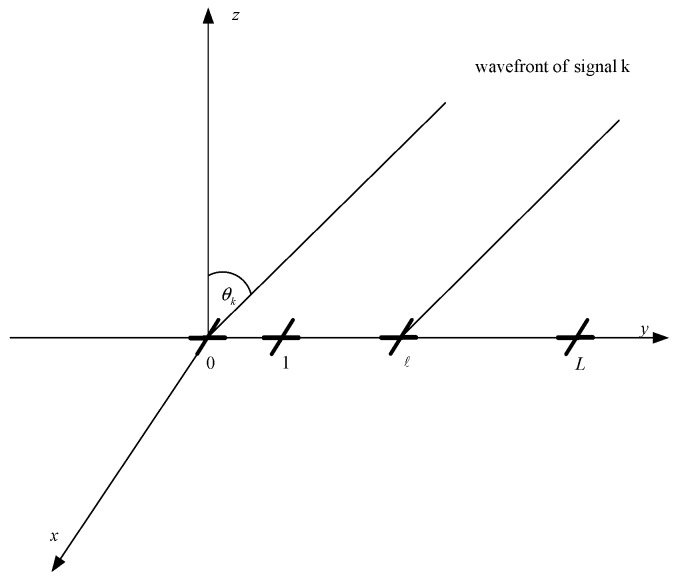
Array configuration.

Assume *K* EM signals are measured by a uniformly linear array (ULA), which is composed of *L* cross-dipoles, as shown in Figure 1. The dipoles are placed along the *y*-axis with inter-spacing d≤λ/2, with *λ* being the wavelength. Also shown in Figure 1, the signals are coming from the directions $\theta_{k},k=1,\cdots ,K$ in the y−z plane. Each dipole pair consists of one *x*-axis dipole, which measures the horizontal polarization component of the signals and one *y*-axis dipole, which measures the vertical polarization component of the signals. Thus, the 2×1 output vector of the *ℓ*th cross-dipole is of the form [23]

(1) $$\begin{array}{c} z_{\ell }\left(t\right)=\sum_{k=1}^{K}q_{\ell }\left(\theta_{k}\right)B_{k}s_{k}\left(t\right)+n_{\ell }\left(t\right) \end{array}$$

where

(2) $$\begin{array}{c} B_{k}=\left[\begin{array}{cc} -1 & 0\\ 0 & \cos\theta_{k} \end{array}\right] \end{array}$$

is the 2×2 response of a cross-dipole due to an EM signal coming from the direction $\theta_{k}$. $q_{\ell }\left(\theta_{k}\right)=e^{-j\left(\ell -1\right)\omega_{k}}$ is the spatial response of the *ℓ*th dipole for the *k*th signal, with $\omega_{k}=2\pi d/\lambda \sin\theta_{k}$. $s_{k}\left(t\right)={\left[\zeta_{k,1}\left(t\right),\zeta_{k,2}\left(t\right)\right]}^{T}$ is the 2×1 signal vector, representing the complex envelopes of the *k*th signal, and $n_{\ell }\left(t\right)={\left[n_{\ell ,1}\left(t\right),\cdots ,n_{\ell ,2}\left(t\right)\right]}^{T}$ is the 2×1 additive noise vector.

The signal covariance matrix of $s_{k}\left(t\right)$ can be written as [23]

(3) $$\begin{array}{ccc} R_{s_{k}} & = & E\{s_{k}\left(t\right)s_{k}^{H}\left(t\right)\}\\ & = & \frac{\sigma_{k,u}^{2}}{2}I_{2}+\sigma_{k,c}^{2}Q\left(\alpha_{k}\right)w\left(\beta_{k}\right)w^{H}\left(\beta_{k}\right)Q^{H}\left(\alpha_{k}\right) \end{array}$$

where

(4) $$\begin{array}{c} Q\left(\alpha_{k}\right)=\left[\begin{array}{cc} \cos\alpha_{k} & \sin\alpha_{k}\\ -\sin\alpha_{k} & \cos\alpha_{k} \end{array}\right] \end{array}$$

(5) $$\begin{array}{c} w\left(\beta_{k}\right)={\left[\cos\beta_{k},j\sin\beta_{k}\right]}^{T} \end{array}$$

with $-\pi /2<\alpha_{k}\leq \pi /2$ and $-\pi /4\leq \beta_{k}\leq \pi /4$ being the polarization orientation angle and polarization ellipticity angle, respectively. $\sigma_{k,u}^{2}$ and $\sigma_{k,c}^{2}$ denote the energy of the unpolarized (UP) part and CP part of the *k*th signal, respectively. The values of $\sigma_{k,u}^{2}$ and $\sigma_{k,c}^{2}$ is used to define the *k*th signal’s degree of polarization (DOP), which is given by $\rho_{k}=\sigma_{k,c}^{2}/\left(\sigma_{k,u}^{2}+\sigma_{k,c}^{2}\right)$. Apparently, $\rho_{k}\in \left(0,1\right)$ corresponds to a PP signal, whose covariance matrix $R_{s_{k}}$ is of full rank, while $\rho_{k}=1$ corresponds to a CP signal, whose covariance matrix $R_{s_{k}}$ becomes rank deficient. Specially, for a CP signal, $s_{k}\left(t\right)$ becomes [24]

(6) $$\begin{array}{c} s_{k}\left(t\right)=\left[\begin{array}{c} \cos\gamma_{k}\\ \sin\gamma_{k}e^{j\eta_{k}} \end{array}\right]\zeta_{k}\left(t\right) \end{array}$$

where $\gamma_{k}$ and $\eta_{k}$ are two polarization parameters, which are, respectively, referred to as the auxiliary polarization angle and polarization phase difference. Hence, we can express the array output vector of a CP signal as

(7) $$\begin{array}{ccc} B_{k}⊗q\left(\theta_{k}\right)s_{k}\left(t\right) & = & B_{k}\left[\begin{array}{c} \cos\gamma_{k}\\ \sin\gamma_{k}e^{j\eta_{k}} \end{array}\right]⊗q\left(\theta_{k}\right)\zeta_{k}\left(t\right)\\ & = & c_{k}⊗q\left(\theta_{k}\right)\zeta_{k}\left(t\right) \end{array}$$

where $c_{k}$ is 2×1 vector containing the polarization parameters of the *k*th CP signal.

Without loss of generality, the first $K_{1}$ signals are assumed to be PP and the remaining $K_{2}=K-K_{1}$ ones are assumed to be PP. Then, using Equations (1) and (7), the 2L×1 array measurements can be expressed in matrix form as

(8) $$\begin{array}{ccc} z(t) & = & A_{\mathrm{CP}}\beta_{\mathrm{CP}}\left(t\right)+A_{\mathrm{PP}}\beta_{\mathrm{PP}}\left(t\right)+n\left(t\right)\\ & = & A\beta (t)+n(t) \end{array}$$

where

(9) $$\begin{array}{ccc} A & = & [\underset{A_{\mathrm{CP}}}{\underset{︸}{c_{1}⊗q\left(\theta_{1}\right),\cdots ,c_{K_{1}}⊗q\left(\theta_{K_{1}}\right)}},\\ & & \underset{A_{\mathrm{PP}}}{\underset{︸}{B_{K_{1}+1}⊗q\left(\theta_{K_{1}+1}\right),\cdots ,B_{K}⊗q\left(\theta_{K}\right)}}] \end{array}$$

is the $2L\times (K_{1}+2K_{2})$ array steering matrix, in which $A_{\mathrm{CP}}$ and $A_{\mathrm{PP}}$, respectively, represent the array steering matrix for CP signals and array steering matrix for PP signals. Although we split the array steering matrix A into two parts in Equation (9), the knowledge of splitting the matrix A into these two matrices is not required in forming the estimators, as will be shown in Section 3.

(10) $$\begin{array}{c} \beta \left(t\right)={\left[\underset{\beta_{\mathrm{CP}}\left(t\right)}{\underset{︸}{\zeta_{1}\left(t\right),\cdots ,\zeta_{K_{1}}\left(t\right)}},\underset{\beta_{\mathrm{PP}}\left(t\right)}{\underset{︸}{s_{K_{1}+1}^{T}\left(t\right),\cdots ,s_{K}^{T}\left(t\right)}}\right]}^{T} \end{array}$$

is the $(K_{1}+2K_{2})\times 1$ signal vector, and

(11) $$\begin{array}{c} q\left(\theta_{k}\right)={\left[q_{1}\left(\theta_{k}\right),\cdots ,q_{L}\left(\theta_{k}\right)\right]}^{T} \end{array}$$

is the L×1 array steering vector for the *k*th signal.

The problem herein is to estimate the angles $\left\{\theta_{k},k=1,\cdots ,K\right\}$ from the *N* snapshots measured at the distinct instants $\left\{t_{n}:n=1,\cdots ,N\right\}$. The polarization state of each signal can also be identified for signal classification purpose. We will propose a conventional MUSIC algorithm based solution to the problem in Section 3, under the following assumptions.

i) The angles $\theta_{1},\theta_{2},\cdots ,\theta_{K}$ are different from each other.
ii) The signal covariance matrix $R_{\beta }=E\left\{\beta \left(t\right)\beta^{H}\left(t\right)\right\}$ is of full rank.
iii) The noise is circularly symmetric complex zero-mean white Gaussian process.
iv) The noise and all the signals are statistically independent.

## 3. The Proposed Method

### 3.1. Determination of Signal Subspace Dimension

As is well known, the performance of the subspace-based methods essentially relies on a priori knowledge of the number of signals. As a result, the first step of the subspace-based methods is to correctly determine the number of signals. In conventional array processing, the number of signals equals to the dimension of signal subspace, which can be estimated by using the Akaike information criterion (AIC) or minimum description length (MDL) criterion, originally proposed in [25]. However, for the problem under consideration, the number of signals ($K=K_{1}+K_{2}$) does not equal the dimension of signal subspace ($K_{1}+2K_{2}$). Processing with AIC or MDL can only obtain the estimates $K_{1}+2K_{2}$. We show in the following subsections that the proposed solution can work properly with the correct estimation of $K_{1}+2K_{2}$, without any knowledge of the numbers of CP signals $K_{1}$, PP signals $K_{2}$, and total signals *K*.

### 3.2. Angle Estimation: PP Signals

First, assume that the dimension of signal subspace $K_{1}+2K_{2}$ is correctly estimated. Then, eigen-decompose the array covariance matrix $R=E\{z\left(t\right)z^{H}\left(t\right)\}$ to construct the $2L\times (K_{1}+2K_{2})$ and $2L\times (2L-K_{1}-2K_{2})$ signal-subspace and noise-subspace matrices E and U, whose columns are, respectively, the 2L×1 eigenvectors associated with the $\left(K_{1}+2K_{2}\right)$ largest and $\left(2L-K_{1}-2K_{2}\right)$ smallest eigenvalues of R. Using the orthogonal property between the signal-subspace, spanned by A, and the noise-subspace, spanned by U, we have

(12) $$\begin{array}{c} U^{H}A=0_{2L-K_{1}-2K_{2},K_{1}+2K_{2}} \end{array}$$

Divide the noise-subspace matrix U as ${\left[U_{1}^{T},U_{2}^{T}\right]}^{T}$, where $U_{1}$ and $U_{2}$ are of the identical sizes. Then, we obtain from Equation (12) that for PP signals,

(13) $$\begin{array}{c} U_{1}^{H}q\left(\theta \right)=U_{2}^{H}q\left(\theta \right)=0,\;\forall \theta =\theta_{k},k=K_{1}+1\cdots ,K \end{array}$$

which gives

(14) $$\begin{array}{c} q^{H}\left(\theta \right)U_{1}U_{1}^{H}q\left(\theta \right)=q^{H}\left(\theta \right)U_{2}U_{2}^{H}q\left(\theta \right)=0\;\forall \theta =\theta_{k},k=K_{1}+1\cdots ,K \end{array}$$

Hence, the angles of the PP signals can be estimated by the spectrum function defined as

(15) $$\begin{array}{c} P_{\mathrm{pp}}\left(\theta \right)={\left[q^{H}\left(\theta \right)\left(U_{1}U_{1}^{H}+U_{2}U_{2}^{H}\right)q\left(\theta \right)\right]}^{-1} \end{array}$$

which is referred to as PP estimator.

*Remark 1*: The PP estimator is quite similar to the conventional MUSIC estimator, except that $U_{1}U_{1}^{H}+U_{2}U_{2}^{H}$, an average version of the noise subspace matrix is used in forming the estimator (For the conventional MUSIC, there is no averaging).

Next, we show that the PP estimator cannot yield the angle estimates of CP signals. For illustration purpose, consider there is only one CP signal. Then, we have

(16) $$\begin{array}{c} U^{H}\left(c⊗q\left(\theta_{1}\right)\right)=0 \end{array}$$

Using the fact that $UU^{H}+EE^{H}=I_{2L}$, we obtain

(17) $$\begin{array}{c} \left[c_{1}^{*}q^{H}\left(\theta_{1}\right),c_{2}^{*}q^{H}\left(\theta_{1}\right)\right]\left[\begin{array}{cc} I_{L}-E_{1}E_{1}^{H} & -E_{1}E_{2}^{H}\\ -E_{2}E_{1}^{H} & I_{L}-E_{2}E_{2}^{H} \end{array}\right]\left[\begin{array}{c} c_{1}q\left(\theta_{1}\right)\\ c_{2}q\left(\theta_{1}\right) \end{array}\right]=0 \end{array}$$

where $c_{1}$ and $c_{2}$ are two polarization components, with $c={\left[c_{1},c_{2}\right]}^{T}$, and $E={\left[E_{1}^{T},E_{2}^{T}\right]}^{T}$, where $E_{1}$ and $E_{2}$ are of the identical sizes.

Rewrite Equation (17), we can obtain

(18) $$\begin{array}{ccc} \underset{\mathrm{equivalent}\;\mathrm{to}\;\mathrm{PP}\;\mathrm{estimator}}{\underset{︸}{|c_{1}{|}^{2}q^{H}\left(\theta_{1}\right)\left(I_{L}-E_{1}E_{1}^{H}\right)q\left(\theta_{1}\right)+{\left|c_{2}\right|}^{2}q^{H}\left(\theta_{1}\right)\left(I_{L}-E_{2}E_{2}^{H}\right)q\left(\theta_{1}\right)}} & = & c_{1}c_{2}^{*}q^{H}\left(\theta_{1}\right)E_{2}E_{1}^{H}q\left(\theta_{1}\right)\\ & + & c_{1}^{*}c_{2}q^{H}\left(\theta_{1}\right)E_{1}E_{2}^{H}q\left(\theta_{1}\right) \end{array}$$

We can observe from Equation (18) that the left side of “=” in Equation (18) is equivalent to the PP estimator, and the right side of “=” in Equation (18) equals $c∥q\left(\theta_{1}\right){∥}_{2}^{4}$, using the fact $E_{1}=q\left(\theta_{1}\right)e_{1}$, $E_{2}=q\left(\theta_{1}\right)e_{2}$, where $c,e_{1},e_{2}$ are non-zero constants. Obviously, ${∥q\left(\theta \right)∥}_{2}^{4}\neq 0$, for any *θ*, and hence, we can obtain from Equations (16) and (18) that $q^{H}\left(\theta_{1}\right)U_{1}U_{1}^{H}q\left(\theta_{1}\right)\neq 0$ and $q^{H}\left(\theta_{1}\right)U_{2}U_{2}^{H}q\left(\theta_{1}\right)\neq 0$. Therefore, we can conclude that the PP estimator will not exhibit peak for an angle corresponding to a CP signal. That is to say, the PP estimator cannot give the angle estimates of CP signals.

### 3.3. Angle Estimation: CP Signals

For CP signals, we obtain from Equation (12) that,

(19) $$\begin{array}{ccc} U^{H}\left(c⊗q\left(\theta \right)\right) & = & U^{H}\left(I_{2}⊗q\left(\theta \right)\right)c=0,\\ & & \forall c=c_{k}\;\mathrm{and}\;\theta =\theta_{k},\;k=1,\cdots ,K \end{array}$$

Let $Q\left(\theta \right)=I_{2}⊗q\left(\theta \right)$. From Equation (19), the MUSIC spectrum for CP signal is given by the following function

(20) $$\begin{array}{c} P\left(\theta \right)={\left[\min_{c}c^{H}Q^{H}\left(\theta \right)UU^{H}Q\left(\theta \right)c\right]}^{-1} \end{array}$$

Generally, the optimization of Equation (20) needs jointly search of both ANGLE and polarization parameters. However, the term in brackets of Equation (20) is minimized by finding the minimum eigenvalue of the 2×2 matrix $Q^{H}\left(\theta \right)UU^{H}Q\left(\theta \right)$. Thus, the angle estimation is decoupled from the polarization estimation, and a joint search of both ANGLE *θ* and polarization parameter c can be avoided. As a result, the MUSIC estimator for CP signal can be expressed as

(21) $$\begin{array}{c} P_{\mathrm{cp}}\left(\theta \right)=\lambda_{\min}^{-1}\left[Q^{H}\left(\theta \right)UU^{H}Q\left(\theta \right)\right] \end{array}$$

which is referred to as CP estimator. In Equation (21), $\lambda_{\min}\left[\cdot \right]$ denotes the minimum eigenvalue operator.

Note that, we can also obtain from Equation (12) that for PP signals,

(22) $$\begin{array}{c} U^{H}\left(I_{2}⊗q\left(\theta \right)\right)=0,\;\forall \theta =\theta_{k},k=K_{1}+1\cdots ,K \end{array}$$

Hence, we get

(23) $$\begin{array}{c} \det\left[Q^{H}\left(\theta \right)UU^{H}Q\left(\theta \right)\right]=0,\;\forall \theta =\theta_{k},k=K_{1}+1\cdots ,K \end{array}$$

where det[·] denotes the determinant operator.

*Remark 2*: From Equations (21) and (23), we can find that the CP estimator Equation (21) also exhibits peaks for angles corresponding to PP signals. However, the PP estimator Equation (15) cannot give the angle estimates of the CP signals. Therefore, a simple method by combining these two estimators for discriminating the CP and PP signals can be formulated as follows. The angles that are obtained from both the CP and PP estimators correspond to the CP signals, while the angles that are estimated from the PP estimator alone correspond to the PP signals. This signal classification method will be introduced in detail in Section 3.4.

*Remark 3*: With the correctly estimated $K_{1}+2K_{2}$, we can obtain the estimation of noise subspace U, and perform PP estimator and CP estimator. Since PP estimator can only give the angle estimates of PP signals, the PP signal number can be found by counting the peaks of PP estimator. We denote ${\hat{K}}_{2}$ as the estimated number of PP signals. At the same time, since CP estimator can give the angle estimates of both CP and PP signals, the total signal number can be found by counting the peaks of CP estimator. We denote $\hat{K}$ as the estimation of total signal number. Then CP signal number can be computed as $\hat{K}-{\hat{K}}_{2}$. Therefore, the proposed method can work properly without any priori knowledge of the numbers of CP signals $K_{1}$, PP signals $K_{2}$, and total signals *K*, as they can be easily found from the formulated estimators.

### 3.4. Signal Classification

As stated in *Remark 2*, signal classification can be achieved by combining the defined estimators. Strictly speaking, in the case of finite samples, the angle estimates of the PP signals estimated from the CP estimator would be different from those estimated from the PP estimator. However, the difference between them is generally much smaller than that of the angular separation, since the MUSIC estimator is asymptotically unbiased, and its bias Δθ satisfies $E\left\{|\Delta \theta |\right\}=O\left(N^{-1}\right)$ [26], where $O(N^{-1})$ denotes in the order of $N^{-1}$. Therefore, the two estimates can be taken as “identical”, if they have sufficiently small difference. In practical, a value that is far smaller than that of the angular separation can be considered as this “sufficiently small” value.

Note also that the implementation of CP estimator is independent to the PP estimator. However, even if the noise is present, the angle association can be obtained automatically from the two sets of the angle estimates (a set of estimates obtained from the PP estimator and another set of estimates obtained from the CP estimator). To see this, suppose $\theta_{1},\cdots ,\theta_{K_{1}}$ are the angles of the CP sources, and $\theta_{K_{1}+1},\cdots ,\theta_{K}$ are the angles of the PP sources. The PP estimator gives the $K-K_{1}$ angle estimates of the PP sources, which are denoted as ${\hat{\theta }}_{1}^{\mathrm{PP}},\cdots ,{\hat{\theta }}_{K-K_{1}}^{\mathrm{PP}}$. The CP estimator yields the *K* angle estimates of both CP and PP signals, which are denoted as ${\hat{\theta }}_{1}^{\mathrm{CP}},\cdots ,{\hat{\theta }}_{K}^{\mathrm{CP}}$. Then, for each $k=1,\cdots ,K-K_{1}$ and ℓ=1,⋯,K, if $|{\hat{\theta }}_{k}^{\mathrm{PP}}-{\hat{\theta }}_{\ell }^{\mathrm{CP}}|<\epsilon_{\theta }$, where $\epsilon_{\theta }$ is a preset small value, which is chosen to be far smaller than the angular separation of the signals, ${\hat{\theta }}_{\ell }^{\mathrm{PP}}$ is associated with a PP signal. Finally, after all the PP angle estimates being picked out from ${\hat{\theta }}_{1}^{\mathrm{CP}},\cdots ,{\hat{\theta }}_{K}^{\mathrm{CP}}$, the remaining angle estimates are associated with the CP signals. Note that the above pairing operation involves minimum computation and requires no additional searches.

*Remark 4*: The proposed method also works for scenarios where all the signals are PP or CP. For example, if both the PP and CP estimators exhibit the same peaks, then the *K* signals are identified as PP. If the PP estimator exhibits no peak, while the CP estimator exhibits *K* peaks, then the *K* signals are identified as CP.

### 3.5. Polarization Estimation for CP Signals

From Equations (12) and (20), for each $k=1,\cdots ,K_{1}$, the polarization parameters of the respective signal can be subsequently estimated by

(24) $$\begin{array}{c} \hat{c}\left(\theta_{k}\right)=v_{\min}\left[Q^{H}\left(\theta_{k}\right)UU^{H}Q\left(\theta_{k}\right)\right] \end{array}$$

where $v_{\min}\left[\cdot \right]$ denotes the eigenvector associated with the minimum eigenvalue.

## 4. Simulation Results

In this section, several numerical simulations are conducted to demonstrate the performance of the proposed method for the problem under consideration. In all the simulations, we consider a ULA composed of L=8 element with d=λ/2. The impinging signals are narrowband, incoherent, equal power, with power defined as $\sigma_{k}^{2}=\sigma_{k,u}^{2}+\sigma_{k,c}^{2}$. The noise is circularly symmetric complex zero-mean white Gaussian process. In each simulation, we perform 500 independent trials.

**Figure 2:**
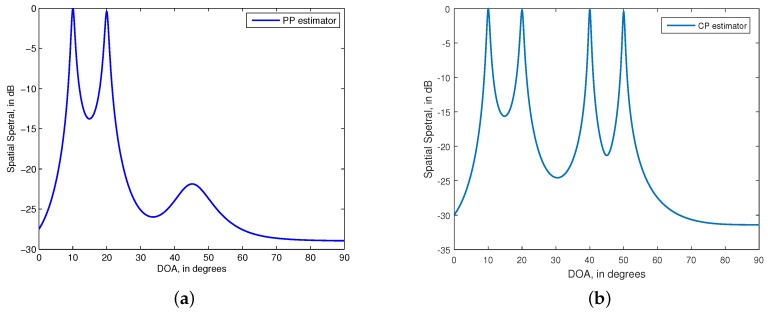
MUltiple SIgnal Classification (MUSIC) spatial spectral of the estimators. (**a**) partially polarized (PP) estimator for estimating two PP signals with $\left\{\theta_{1},\theta_{2}\right\}$ = $\{10^{\circ },$ $20^{\circ }\}$; (**b**) completely polarized (CP) estimator for estimating two CP signals with $\left\{\theta_{3},\phi_{4}\right\}$ = $\{40^{\circ },$ $60^{\circ }\}$. SNR=10 dB, N=200. 500 independent trials.

In the first experiment, we assume two PP signals and two CP signals. The arrival signals of the signals are $\{\theta_{1},\theta_{2},$ $\theta_{3},\theta_{4}\}$ = $\{10^{\circ },$ $20^{\circ },\;40^{\circ },$ $50^{\circ }\}$. The DOP of the two PP signals are identical and equal to 0.5. The SNR and snapshots are, respectively, set as 10dB and N=200. The MUSIC spectral of the PP estimator and CP estimator are given in Figure 2. We see from the figure that the CP estimator can provide the angle estimates of both the PP and CP signals, while the PP estimator can only give the angle estimates of the PP signals. This phenomenon is in agreement with the discussion in Section 3. We can conclude from Figure 2 that the first two signals are PP and the last two signals are CP.

**Figure 3:**
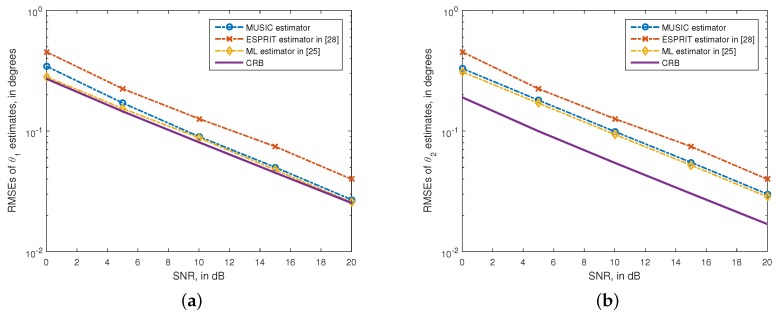
RMSEs of $\theta_{1}$ and $\theta_{2}$ estimates *versus* SNR. (**a**) $\theta_{1}=10^{\circ }$; (**b**) $\theta_{2}=20^{\circ }$, $\rho_{2}=0.5$. N=200. 500 independent trials.

In the second experiment, the scenario of coexistence of one PP signal and one CP signal is considered. We assume the first is CP with $\theta_{1}=10^{\circ }$, and the second is PP with $\theta_{2}=20^{\circ }$ and $\rho_{2}=0.5$. The data samples used in simulations are N=200. The RMSEs of the angle estimates *versus* the SNR are given in Figure 3. In Figure 3, we also plot the the RMSEs of ESPRIT estimates [24], ML estimates [21] and the stochastic CRBs for comparison. We can see from Figure 3 that, the RMSEs of the proposed method are smaller than those of the ESPRIT algorithm, and they are close to the CRBs. Moveover, the RMSE difference between the proposed method and the ML algorithm is very small.

**Figure 4:**
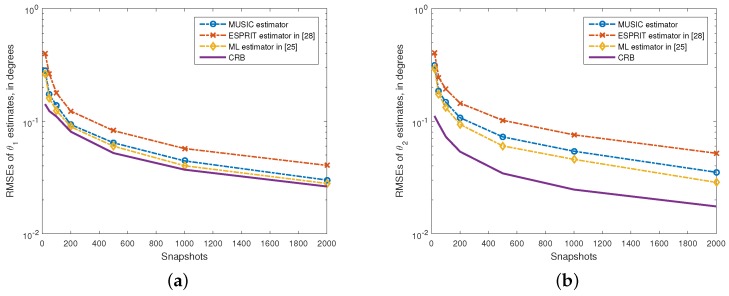
RMSEs of $\theta_{1}$ and $\theta_{2}$ estimates *versus* the number of snapshots. (**a**) $\theta_{1}=10^{\circ }$; (**b**) $\theta_{2}=20^{\circ }$, $\rho_{2}=0.5$. SNR = 10 dB. 500 independent trials.

In the third experiment, the performance of the proposed method against the number of snapshots is assessed. The SNR is set as 10dB and the snapshot numbers are varied from N=20 to N=2000 in this experiment. The remaining parameters used is the same as those used in the second experiment. We illustrate the RMSEs of the angle estimates in Figure 4, where the RMSEs of ESPRIT and ML estimates and CRBs are also given for comparison. It is obvious that the proposed method provides angle estimates that are more accurate than that provided by the ESPRIT-based method in [24], with a much smaller RMSE. Moreover, the RMSEs of all the algorithms drop monotonically with the number of snapshots. In addition, the performance of the proposed method is very close to that of the ML algorithm.

## 5. Conclusions

We have presented a MUSIC-based method for angle estimation and signal classification under the simultaneous existence of completely polarized and partially polarized electromagnetic signals. We have defined two estimator to estimate the angles of CP and PP signals separately. We have also presented a simple method for signal classification by combining the defined two estimators. The proposed method is efficient since it does not require a multidimensional search or the estimation of the degree of polarization (DOP) of the signals. Incidentally, the proposed method offers a performance very close to that of the ML algorithm, but with a much reduced computational costs in that no multidimensional search is required. To effective application of MUSIC, the proposed method requires the number of sensors $L>K_{1}+2K_{2}$.
